# New Perspectives Related to the Bioluminescent System in Dinoflagellates: *Pyrocystis lunula*, a Case Study

**DOI:** 10.3390/ijms21051784

**Published:** 2020-03-05

**Authors:** Carlos Fajardo, Marcos De Donato, Hectorina Rodulfo, Gonzalo Martinez-Rodriguez, Benjamin Costas, Juan Miguel Mancera, Francisco Javier Fernandez-Acero

**Affiliations:** 1 Microbiology Laboratory, Institute of Viticulture and Agri-food Research (IVAGRO), Environmental and Marine Sciences Faculty. University of Cadiz (UCA), 11510 Puerto Real, Spain; carfaqui07@yahoo.es; 2 Tecnologico de Monterrey, Escuela de Ingenieria y Ciencias, 76130 Queretaro, Mexico; mdedonate@tec.mx (M.D.D.); herodulfo@tec.mx (H.R.); 3 Institute of Marine Sciences of Andalusia (ICMAN), Department of Marine Biology and Aquaculture, Spanish National Research Council (CSIC), 11519 Puerto Real, Spain; gonzalo.martinez@csic.es; 4 Interdisciplinary Centre of Marine and Environmental Research of the University of Porto (CIIMAR), 4450-208 Matosinhos, Portugal; bcostas@ciimar.up.pt; 5 Institute of Biomedical Sciences Abel Salazar (ICBAS-UP), University of Porto, 4050-313 Porto, Portugal; 6 Faculty of Marine and Environmental Sciences, Biology Department, University of Cadiz (UCA), 11510 Puerto Real, Spain; juanmiguel.mancera@uca.es

**Keywords:** luciferase, luciferin-binding protein, luciferin, P630, blue compound, glutathione S-transferase

## Abstract

*Pyrocystis lunula* is considered a model organism due to its bioluminescence capacity linked to circadian rhythms. The mechanisms underlying the bioluminescent phenomenon have been well characterized in dinoflagellates; however, there are still some aspects that remain an enigma. Such is the case of the presence and diversity of the luciferin-binding protein (LBP), as well as the synthesis process of luciferin. Here we carry out a review of the literature in relation to the molecular players responsible for bioluminescence in dinoflagellates, with particular interest in *P. lunula*. We also carried out a phylogenetic analysis of the conservation of protein sequence, structure and evolutionary pattern of these key players. The basic structure of the luciferase (LCF) is quite conserved among the sequences reported to date for dinoflagellate species, but not in the case of the LBP, which has proven to be more variable in terms of sequence and structure. In the case of luciferin, its synthesis has been shown to be complex process with more than one metabolic pathway involved. The glutathione S-transferase (GST) and the P630 or blue compound, seem to be involved in this process. In the same way, various hypotheses regarding the role of bioluminescence in dinoflagellates are exposed.

## 1. Introduction

Dinoflagellates are the most important eukaryotic protists that produce light [1,2]. This singularity has inspired not only literature and art, but also an intensive scientific dissection [3,4,5]. *Pyrocystis* has been a main model genus for a long time in the study of bioluminescence in dinoflagellates [6,7,8,9,10] as well as in the development of some biotechnological applications associated with its bioluminescence capacity [11,12,13].

All dinoflagellates belong to the Dinophyceae group and have been unchallengeably placed using extensive molecular phylogenetic data within the Alveolata group, being closely related to the Apicomplexa group, which includes many parasitic species [14]. *Pyrocystis* (Dinophyceae) spends a large part of its life as a non-mobile cell on a shell covered with cellulose [15,16]. *Pyrocystis* includes a small number of marine species that have a cosmopolitan distribution [17]. The life cycles of *P. lunula*, as in other species of this genus, it is characterized by a normal asexual reproduction linked to simple alternations of coccoid cells and morphologically different transitory reproductive stages. There are different reproductive bodies depending of the species. In the case of *P. lunula*, the reproductive bodies are athecate-uniflagellate planospores. In *P. noctiluca* and *P. fusiforrnis* are athecate aplanospores. For *P. lanceolate* are athecate-biflagellate, and in *P. acuta* are thecate-biflagellate [18]. Furthermore, evidence of sexual reproduction has been reported in *P. lunula* [19]. The *P. lunula* lifestyle is also characterized by the execution of vertical migrations in relation to the circadian rhythm [20].

The bioluminescent light is generated by a chemical reaction. Although the process is not the same in all the bioluminescent organisms, most of them share the same base reaction; where the LCF enzyme reacts with the luciferin (substrate) in presence of oxygen and produces an oxyluciferin that emits a photon while it decays from a high to a low energy state. There are exceptions to this base reaction, for example, in some luminous earthworms [5] and acorn worms [21], the bioluminescent event is triggered by H_2_O_2_ and not for O_2_. Besides, the electronic structures, absorption and fluorescence spectra of luciferin, its six analogues and its oxidized form, oxyluciferin showed clear evidence of the lack of fluorescence in *Latia neritoides* [22].

The cellular mechanisms and the genes involved with bioluminescence in dinoflagellates are well characterized. The bioluminescence system in these organisms is unique, from a molecular and cellular point of view. The production of light takes place in specialized organelles, the scintillons, which contain the LCF enzyme, the substrate luciferin, and in most cases LBP [23,24,25,26]. The light emission is based on LCF-catalyzed oxidation of the luciferin, generally protected from oxidation by LBP that binds the luciferin at physiological pH. Furthermore, molecular studies have demonstrated a high variation in the sequences of LBP, showing a highly diverse gene family including several non-identical copies arranged in tandem within the genome [27,28], like in *Lingulodinium polyedra* [27,29,30], *Noctiluca scintillans* [31], *Alexandrium* spp. [32,33,34] and *Pyrocystis lunula* [35]. The LBP has also been found in the genera *Gonyaulax*, *Ceratocorys*, *Protoceratium* [36,37].

Until recently, it was thought that the genus *Pyrocystis* was among the few ones lacking the presence of the LBP [23,25,37] such as in genera *Ceratium*, *Fragilidium*, and *Protoperidinium* [37]; however, this fact was refuted by the recent detection and characterization of LBP in *P. lunula* [30]. Emerging information shows substantial evidence that LBP is an integral component of the standard molecular bioluminescence system in dinoflagellates [35,37,38].

Another important fact that today remains an enigma refers to what is the exact mechanism underlying the luciferin synthesis process. In *P. lunula*, in contrast to other bioluminescent dinoflagellates, the levels of LCF and luciferin are constant throughout the daily cycle [25]. Therefore, the rhythm is related to changes in their intracellular localization, instead of daily *de novo* synthesis and destruction of all the components [16,39]. According to available evidence, it has been proposed that luciferin can be synthesized through different ways, and is thought to be universal in dinoflagellates, because luciferin from any dinoflagellate bioluminescent species can be used it as subtract to produce light [40]. It was suggested that luciferin is a photo-oxidation breakdown product of chlorophyll a [41]; however, this would not be true in all cases since *L. polyedra* only contains luciferin during the night period when photo-oxidation is not possible; and *Protoperidinium crassipes* can preserve even one year its bioluminescence in the absence of food with chlorophyll or luciferin [42] and, therefore, it can be suggested that it contains luciferin originated from a different precursor. It is likely that more than one mechanism is responsible for luciferin production [38]. In fact, a study with amino acid tracers has confirmed the intracellular production of luciferin [43], and in this regard, Fresneau and Arrio [44] argue that bioluminescence in dinoflagellates is ruled by the reduction state of the luciferin precursor. Regarding the controversies in relation to these issues, we have made a bibliographic search and metanalysis that explores the current available knowledge in relation to the function of bioluminescence in dinoflagellates and the description of the classic components of the system, such as the LCF. It also gives some new perspectives regarding the phylogenetic diversity of LBPs and the process of synthesis of luciferin, and therefore of the bioluminescent mechanism underlying these organisms. Due to the ecological importance of Dinophyceae in marine environments and to the bioluminescence as a strategy for competition and/or survival, we carry out, on one hand, a comprehensive literature review to compile all the knowledge about the key players involved in the production of bioluminescence in dinoflagellates, and, on the other hand, we carry out a phylogenetic analysis of the conservation of protein sequence, structure and evolutionary pattern of these key players.

## 2. Phylogenetic and Structural Analyses

Sequences of the dinoflagellate species for the genes LCF, LBP and GST were downloaded from GenBank (Table 1). Sequences were aligned using MUSCLE software [45] and the phylogenetic analysis was carried out using the Maximum Likelihood method based on the General Time Reversible model [46] using MEGA software v 7.0.14 [47] with 1000 bootstrap value [48]. A discrete Gamma distribution was used to model evolutionary rate differences among sites (5 categories). The prediction of the 2D/3D structure of the conserved regions analyzed, were carried out using the Phyre2 web server [49] (www.sbg.bio.ic.ac.uk/~phyre2/html/page.cgi?id=index) and the resulting structures were visualized with the molecule modelling software EzMol (version 1.22, www.sbg.bio.ic.ac.uk/ezmol) [50]. Sequence logo was made using WebLogo (weblogo.berkeley.edu).

## 3. Dinoflagellate Bioluminescent System

**Figure 1:**
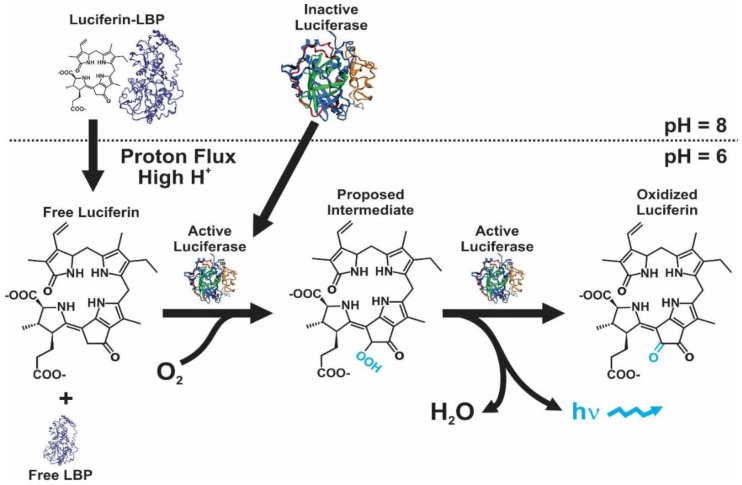
Bioluminescence model in dinoflagellates, showing the effect of pH on both LBP and LCF. Modified from the proposed model by Rüdiger Hardeland (http://tolweb.org/notes/?note_id=5621) and work published by Morse et al. [30]. The structure of LBP is not known, the one shown here it was obtained by using the *P. lunula* sequence into Phyre2 and it is shown for illustration purposes only. The structure of LCF shown here was predicted in Phyre2 using the *P. lunula* sequence, as explain in the text and in Figure 2.

Bioluminescence has been reported within dinoflagellates only in marine species in approximately 6% of all genera [51]. This system is similar between species regarding the cross-reactions between enzymes and substrates, pH activity profiles and their cellular location in the scintillons, the light-emitting organelles [23,52]; however, as with all bioluminescence systems, this is unique from a molecular and cellular perspective. The scintillons [53] contain the luciferin, the LCF and, in some species, LBP [23,24,25]. The scintillons are organelles [24,52] which, during dark hours, are distributed around the periphery of the cell [16]. A flash of light is induced by an action potential along the membrane of the vacuole, trigger by a mechanical stimulus of the cell, and involving of a voltage-dependent proton channel [54,55]. This produce a reduction in pH (i.e., from 8.0 to near 6.0) within the scintillons, which activates the LCF and causes the LBP to release the luciferin (Figure 1), making it available for oxidation by LCF [29]. After full stimulation during the dark period, a single cell of *P. lunula* emits approximately 4 × 10^9^ photons, which is an order of magnitude greater than the light emitted by *Pyrodinium bahamense* and *L. polyedra* [37,56].

Light emission occurs in different ways. The flashing consists of brief (0.1 s) and intense light (peak intensity, ~10^9^ quanta s-1 cell-1). Cells can also emit a low intensity emission that gradually rises to a peak (~10^4^ quanta s-1 cell-1), and decrease to zero until the end of the night. The total quantity of light produced by the glow each day is about 10^7^ quanta cell-1 [57]. How the circadian regulation of the bioluminescence in *P. lunula* is accomplished is unknown; nevertheless, it was reported that the localization of the scintillons differs during the daily cycle [9,58,59]. In this case, the scintillons are relocated in relation to chloroplasts in an interchangeably way, so that during the night the scintillons are in the periphery of the cell and migrate to the center in the day. In this way, by preventing stimulation, they can modulate the bioluminescent intensity during the day [19,60]. Bioluminescence represents one of the fastest mechano-sensitivity systems known to date, as the delay between stimulus and response is only of 15–20 ms [61,62,63,64].

### 3.1. Luciferase (LCF)

**Figure 2:**
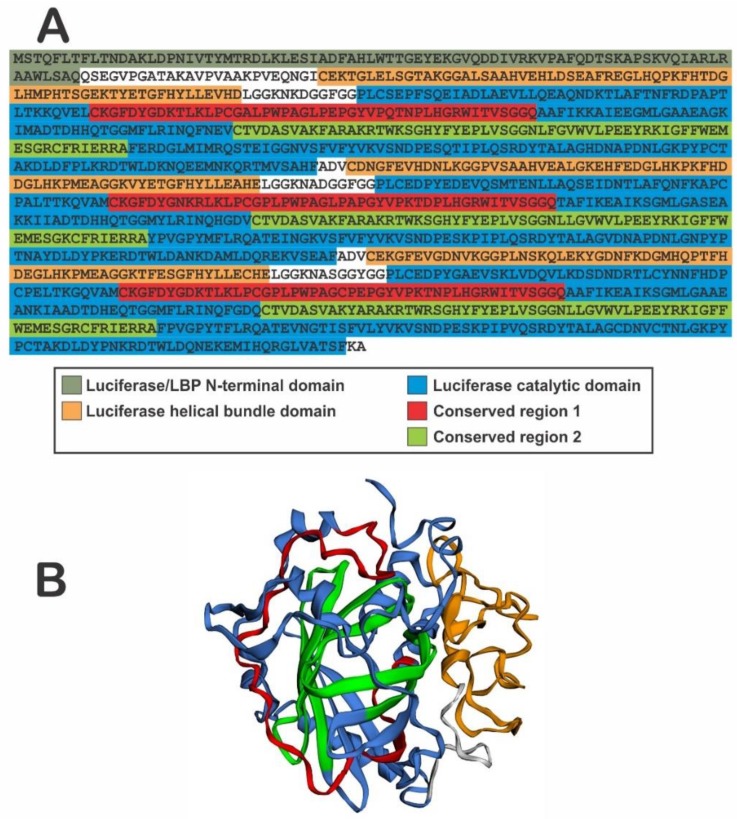
(**A**) Sequence of *P. lunula* LCF protein A (GenBank AAL40676) showing the Luciferase/LBP N-terminal, helical bundle and catalytic domains, as well as the two conserved regions within the catalytic domain. (**B**) Our prediction of the 2D/3D structure of the first helical bundle (yellow) and first catalytic domain of the LCF (blue) of *P. lunula* (GenBank AAL40676), showing the conserved regions 1 (red) and 2 (green), made with Phyre2 web server and visualized with EzMol, as described in the text.

Dinoflagellate LCF catalyzes the oxidation of luciferin by molecular oxygen, originating an electronically excited oxyluciferin that emits blue light at λmax of 480 nm. In *P. lunula*, as in all the Gonyaulacales studied so far, LCF (MW = 137 kDa) has a single luciferase/LBP N terminal domain and 3 catalytic domains, preceded by helicase bundle domains (Figure 2), with each catalytic domain, of approximately 46 kDa, being enzymatically active, and its coding sequence is arranged, within the genome, in several copies in tandem [65,66]. In *P. lunula* there are three different isoforms of the LCF gene: LCFa (GenBank AF394059.1), LCFb (GenBank AF394060.1), and LCFc (GenBank AF394061.1) [34], and the primary structure is highly conserved [67].

**Figure 3:**
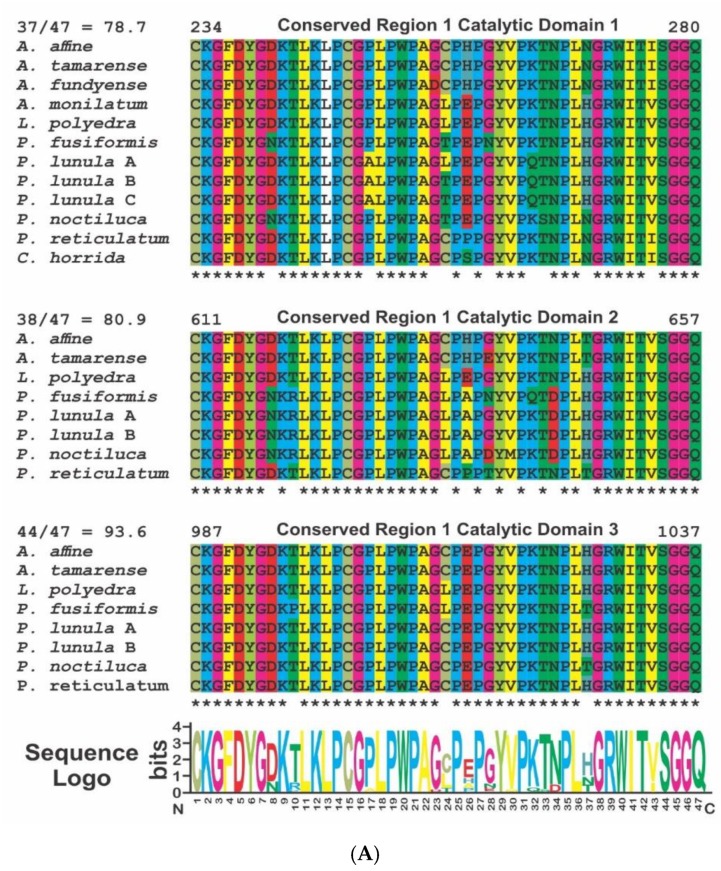

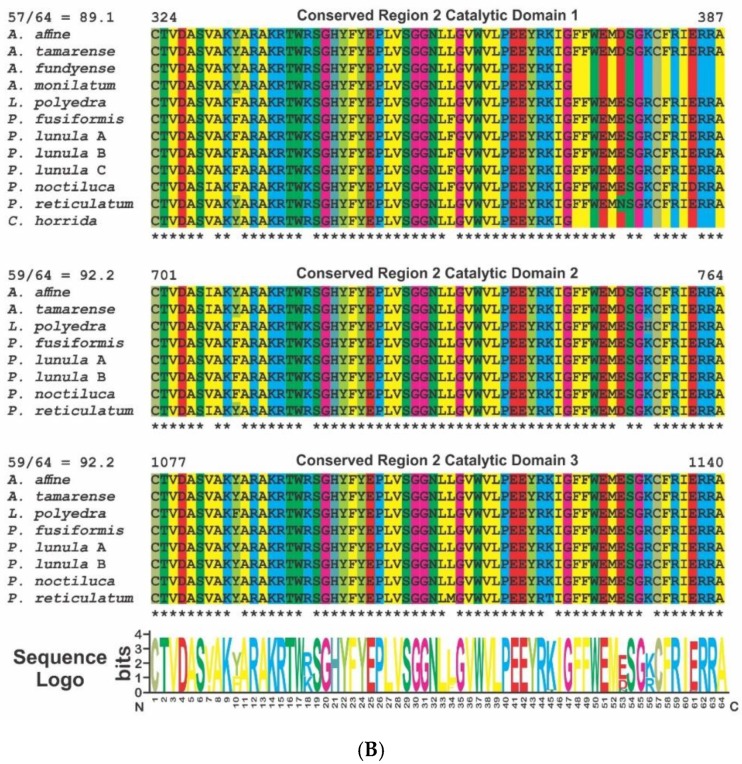
Sequence alignment of the conserved regions 1 (**A**) and 2 (**B**) found in the LCF comparing across the three catalytic domains and the available sequences from the species of the genera *Alexandrium*, *Lingulodinium*, *Pyrocystis* and *Protoceratium*. Numbers on top of each region correspond to the amino acid position in *P. lunula* protein (GenBank AAL40676). Asterisks are showing the conserved amino acids in each position. Coloring of the amino acids was made according to the same pattern displayed in MEGA7 software. Sequence logo was made using WebLogo, as described in the text.

**Figure 4:**
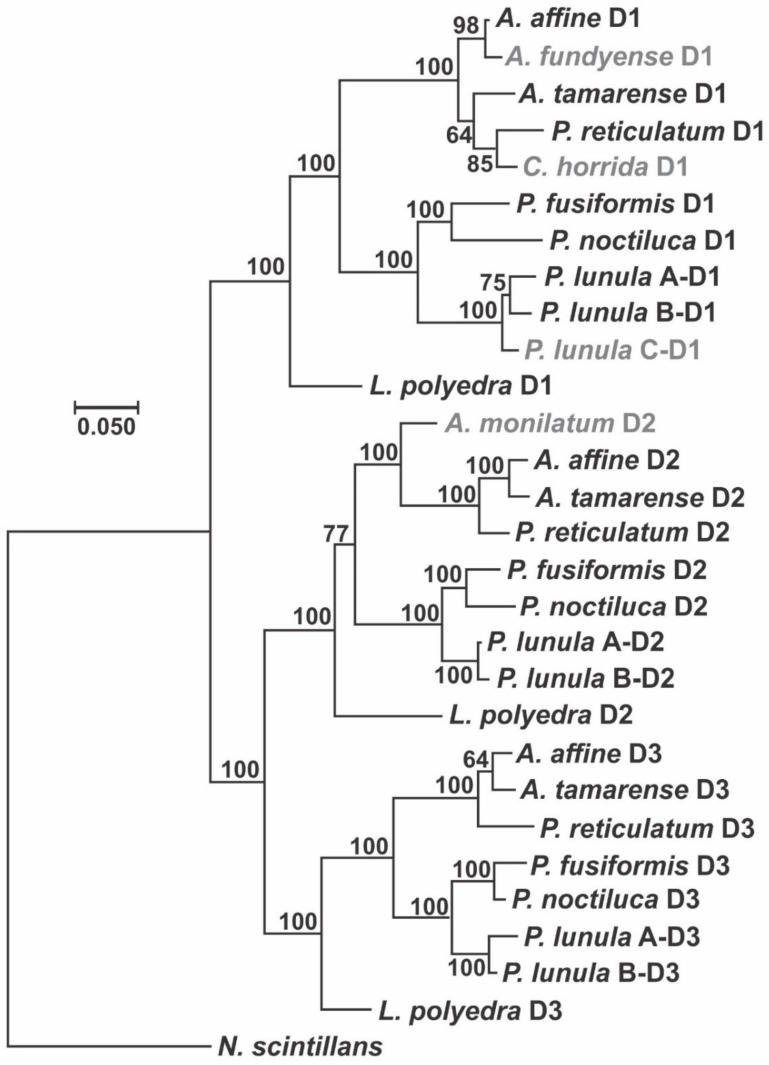
Molecular Phylogenetic analysis by a Bayesian method, showing the evolutionary pattern of the three catalytic domains in the species of dinoflagellates with protein sequences available, using the sole catalytic domain from *N. scintillans* as outgroup. The percentage of trees in which the associated taxa clustered together is shown next to the branches. There was a total of 297 positions in the final dataset and the tree is drawn to scale, with branch lengths measured in the number of substitutions per site.

**Figure 5:**
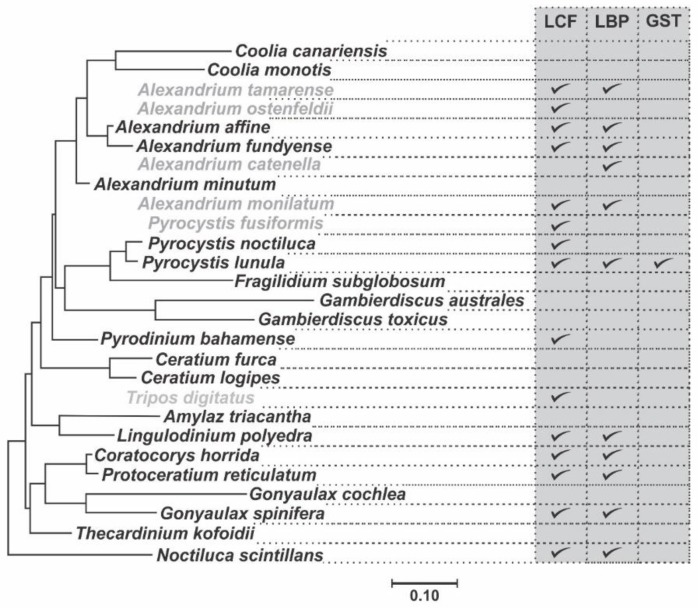
Phylogenetic tree of Gonyaulacales among the dinoflagellates inferred from the three rDNA, two mitochondrial and four nuclear protein genes, modified from Orr et al. (2012). The tree is reconstructed with Bayesian inference (MrBayes) and included the different groups within dinoflagellates, but here only the Gonyaulacales are shown. Species in gray were not included in the original analysis by (Orr et al. 2012). The table is showing which of these species have reported LCF, LBP and GST protein sequences.

The alignment of the sequences of all three catalytic domains in the available species of Gonyaulacales shows the presence of 2 highly conserved domains (Figure 3) that should be associated to the catalytic function of the enzyme. The sequence logo shows 87.2 and 93.8 identity in the amino acid sequences in regions 1 and 2, respectively. The phylogenetic analysis of the LCF domains of the species of Gonyaulacales, using LCF from *N. scintillans* as an outgroup, which only contains one catalytic domain, shows an evolutionary pattern very similar for each of the three domains (Figure 4). The phylogenetic relationship among the species is conserved but has some differences to a previously publish study (Figure 5) [68], since the tree presented here shows a closer relationship between the species of the genus *Alexandrium* and *P. reticulatum* and *C. horrida* than to the genus *Pyrocystis*, which is the opposite to what is reported in the phylogenetic relationship built using the sequences of 8 different genes (28S, 5.8S, 18S, cox1, cob, beta-tubulin, actin, and hsp90). It is likely that the formation of the three domains occurred by duplication of domain 1 and before the diversification of the Gonyaulacales, as is a common feature in all of the sequenced species, but there is no evidence if the three domains are present outside of the Gonyaulacales, since there is no report of the full LCF sequence in any other dinoflagellate outside this order, other than *N. scintillans*.

**Figure 6:**
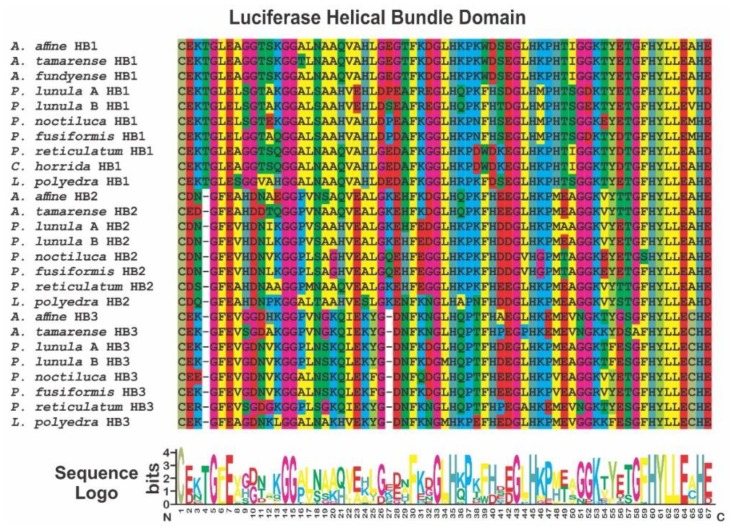
Sequence alignment of the three LCFs helical bundle domains contained in species of the genera *Alexandrium*, *Lingulodinium*, *Ceratocorys*, *Pyrocystis* and *Protoceratium* that have their protein published in GenBank. Coloring of the amino acids was made according to the same pattern displayed in MEGA7 software. Sequence logo was made using WebLogo, as described in the text.

In *L. polyedra*, the activity of LCF was reported to be maximum at pH 6.3, decreasing at pH 8 (almost to zero). Four intra-molecularly conserved histidines in the LCF helical bundle domain are linked to the loss of activity at high pH [69]. These histidine residues are also conserved in *P. lunula* [39,65,66,67,70], as well as in the other species analyzed here, as are the whole helical bundle domains (Figure 6). The first two of the conserved histidines are preceded by glycine and leucine and the last two are in a highly conserved region formed by ten amino acids.

The structure of the 3rd domain of *L. polyedra* LCF was reported [71], and the general structure can be divided in two main parts: 1) the barrel-like, that compresses the core of the enzyme and has the active site where the oxidation of luciferin and light production occur, and 2) the lid of the barrel, which is closed by a three-helix bundle that acts like a regulatory structure at the top. This structure is very similar to the one reported here as a prediction for *P. lunula* LCF catalytic domain (Figure 2), with the conserved regions 1 and 2 located at the walls of the barrel, which suggest that it is likely to be a good approximation of the real structure. Molecular dynamics studies suggest that once the pH drops near 6, the regulatory N-terminal histidines begin a conformational change in the three-helix bundle [72]. The lid of the barrel opens and admit the access of luciferin to the active site, where it is oxidized. In this structure, water molecules are occupying the active site that would be taken by the luciferin during the bioluminescent event. The N-terminal histidine residues that control the activity by pH are at the link between the helices in the bundle. The pKa of the histidine generally ranges 6-6.5, which indicates that the protonation state of these residues possible change when the pH drops below 6.5, where LCF is active. For LCF, the activity is regulated by the protonation state of the histidine residues located outside the active site [72].

Recently, Donnan et al. [73] applied Constant pH Molecular Dynamics to study the structural changes linked with the activation of LCF upon acidification. The protonation of some residues, including the previously reported intra-molecularly conserved histidines, and the H1064/H1065 dyad (inside the catalytic domain), correlates with a large-scale structural change in which the helical bundle domains are regrouped to allow luciferin access to the active site. In parallel, the β-barrel expands and a putative active site base, E1105, takes into position starting the catalysis reaction.

PCR using genomic DNA of *P. lunula* showed that both LCFa and LCFb but not LCFc are in tandem repeats, but there is no identifiable promoter in the intergenic spacers, which has led to the suggestion that tandem gene repeats may form a polycistronic transcript, very similar to the reported in the case of *Trypanosoma* [38,74]. This could increase the efficiency of the transcription/translation of LCF.

In some cases, like in *L. polyedra*, the abundance of LCF protein shows a circadian rhythm, being synthesized in the early night and destroyed in the course of the day. Nevertheless, there are clear differences in the *P. lunula* system, where flicker but not brightness has a circadian rhythm. Moreover, the quantities of luciferin and LCF are constant during the day and night in *P. lunula* [9,25]. In relation to why a mechanism of *de novo* synthesis of the molecular components of the bioluminescent system exists in some species, such as *L. polyedra*, which is somehow unexpected having in mind the energy expenditure involved, Hasting [20] suggested that it may represent a mechanism to conserve nitrogen. For instance, amino acids released from the hydrolysis of LBP and LCF, could be available for the biosynthesis of other proteins over the course of the circadian cycle [20].

In the case of *P. lunula*, which also exhibit a circadian rhythm of bioluminescence but do not destroy and resynthesize LCF, the mechanism may be related to the ecology of this species as they make a daily vertical migration to deeper waters, where nitrogen is more available. It is also presumed that the high number of copies of LCF resulted from an increase in the requirement of a large quantity of this protein, which exercises a selective pressure for the retention of duplicate genetic copies [70,75]. Another possible reason for the presence of three active catalytic sites on a single molecule could be related to an enhancement in LCF activity without an increase in the colloidal osmotic pressure inside the scintillon [72].

### 3.2. Luciferin-Binding Protein (LBP)

*L. polyedra* represents the model organism for studies with LBP [27,30,76]. In this species, LBP has been found to be very abundant (up to 1% of the total proteome) [30]. One isoform of the protein sequence contains a LCF/LBP N-terminal domain (pfam05295, Luciferase_N), highly similar to the one found in LCF. It has been suggested that this region may mediate an interaction between LBP and LCF or their association with the vacuolar membrane [37]. It does not appear to be a signal or transit peptide, and LCF and LBP do not transit through the membrane in the formation of the scintillons [77]. Scintillons are formed near the Golgi, where the two proteins are associated before migrating together to the vacuolar membrane [72].

In *L. polyedra*, LBP is represented by two gene types which share a high sequence identity and that encode for two proteins expressed in the same levels [27,30]. Multiple isoforms of genes related to bioluminescence are common in LBP and LCF in some Gonyaulacales species [27,39,67]. Furthermore, each gene type is present in different copies arranged in tandem, originating more divergence among this gene family [27,28]. Published research [35,37] have shown the diversity and distribution of LBP in several Gonyaulacales, such as *A. affine, A. fundyense, A. monilatum, A. catenella, A. tamarense, Ceratocorys horrida, Gonyaulax spinifera, Protoceratium reticulatum,* and *L. polyedra* (Figure 5).

**Figure 7:**
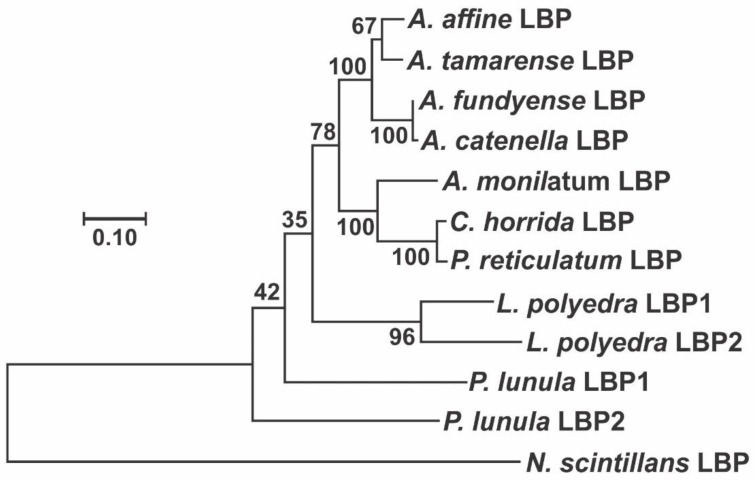
Molecular Phylogenetic analysis by a Bayesian method, showing the evolutionary pattern of LBP from the species of the genera *Alexandrium*, *Lingulodinium*, *Pyrocystis* and *Protoceratium* that have their protein published in GenBank, using the sequence from *N. scintillans* as outgroup. The percentage of trees in which the associated taxa clustered together is shown next to the branches. There was a total of 435 positions in the final dataset and the tree is drawn to scale, with branch lengths measured in the number of substitutions per site.

The phylogenetic analysis of the LBP sequences available in GenBank shows an evolutionary pattern very similar to that obtained in the analysis of the catalytic domains of LCF (Figure 7), differing from the pattern seen in the Gonyaulacales (Figure 5), when analyzing 8 different genes (28S, 5.8S, 18S, cox1, cob, beta-tubulin, actin, and hsp90) [68]. This analysis shows LBP sequences from *C. horrida* and *P. reticulatum* being very closely related to the species of *Alexandrium*, while the sequence from *P. lunula* seems to be the most divergent.

In the case of *Pyrocystis*, it was reported to lack expressed LBP [23,25]. Nevertheless, a recent transcriptome and proteome study reported two different types of LBP being present in *P. lunula* [35], one that correspond with the individual gene LBP (GenBank MN259726), highly similar to *A. tamarense* (GenBank AFN27006.1) [37], and another that represent a gene fusion between LCF/LBP (GenBank MN259727), similar to *L. polyedra* (GenBank AAA29164.1 and AAA29163.1) [27] and *A. cantenella* (GenBank ABY78836.1) [33].

LBP sequences have shown to be a very large and highly diverse gene family [27,28] and despite many efforts, the differences in the sequences between species has precluded the design of universal primers for LBP, as previously reported for LCF [67]. In a similar way, on *Alexandrium* spp., LBP sequences showed more than one type of this protein, which is in line with previous observations of their LCF gene sequences [67]. Studies in *N. scintillans* also showed that LBP is present in diverse forms and as a result of important evolutionary events like as fission or fusion of genes. The combined LCF/LBP gene previously reported for this organism [31,37], consist of part of the LCF N-terminal domain and the LCF catalytic domain followed by the LBP domain. The characteristic element of the hybrid LCF/LBP is the presence of the LCF/LBP N-terminal domain present in the separate genes of LCF and LBP in the photosynthetic species.

These sequences, which shared the LBP domain and the N-terminal region, but not the LCF domain, corresponded to 2 different genes: a single separated LBP, and the combined LCF/LBP. In the case of *N. scintillans*, it was confirmed the expression of both genes [37]. It has been suggested that a second LBP could have another related function, either by binding luciferin in the scintillons or in the cytoplasm; however, this is a hypothesis that still needs to be unraveled [31]. In case that LCF/LBP of *N. scintillans* is ancestral to the separated LBP and LCF in photosynthetic species, as suggested by their phylogeny [68], the single LBP of *N. scintillans* could have been originated by mRNA splicing off the LCF/LBP gene and later was inserted by retro transposition in the genome, condition that was acquired by the modern species [78,79]. The N-terminal region in these genes suggest this hypothesis.

**Figure 8:**
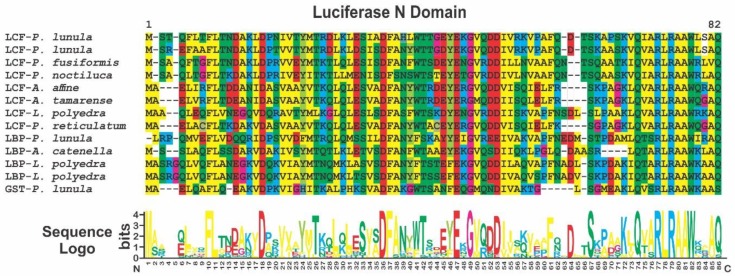
Sequence alignment of the luciferase/LBP N terminal domain contained in the LCF, LBP and GST from the species of the genera *Alexandrium*, *Lingulodinium*, *Pyrocystis* and *Protoceratium* that have their protein published in GenBank. Coloring of the amino acids was made according to the same pattern displayed in MEGA7 software. Sequence logo was made using WebLogo, as described in the text.

Having separate genes for LBP and LCF could allow a differential regulation. This can represent an advantage as LCF has a triple catalytic capacity in the modern photosynthetic species [37], while LBP needs to be proportional to luciferin (stoichiometrically) [29]. The LCF N terminal domain shows high conservation between the LCF and LBP proteins and among the photosynthetic species with available sequences (Figure 8), highlighting the important role that this domain has for the function of both proteins in the bioluminescence system. Interesting is the high frequency of both charged and non-charged polar amino acids, which could suggest a role in the interaction of these proteins with the polar surface of the phospholipids in the membranes.

### 3.3. Luciferin

**Figure 9:**
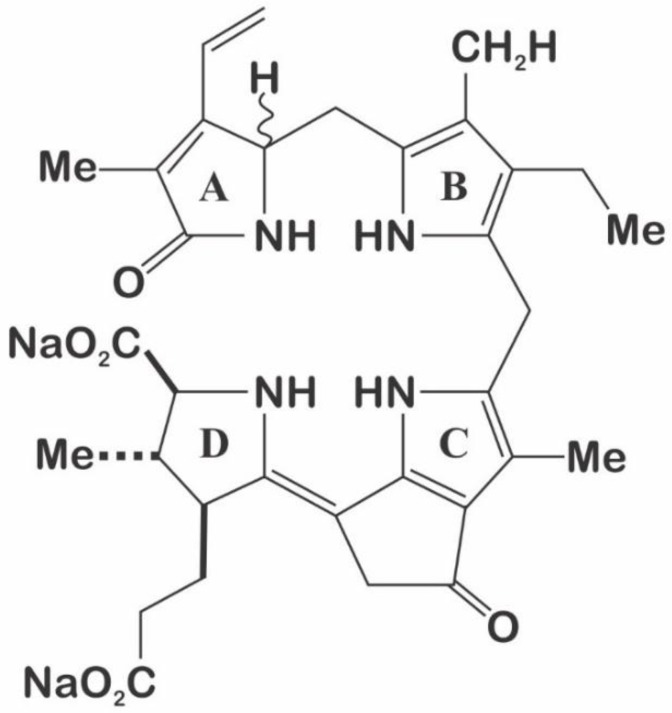
Structure of the dinoflagellate luciferin, according to Wang and Liu, (2017).

*P. lunula* luciferin is a tetrapyrrole-type molecule (Figure 9), similar to chlorophyll a and euphausiid shrimp luciferin (*Euphasia superba*) [40,80]. This luciferin is extremely labile to oxidation, photo-oxidation at high salt concentration, and at low pH [81]. *P. lunula* has been reported to contain larger quantities of luciferin than any other dinoflagellate [45], even 100 times more than *L. polyedra* [25,80]. Furthermore, luciferin purified from *P. lunula* can cross-react with the LCFs of many other bioluminescent dinoflagellate species and could even cross-react with the bioluminescent system of the krill *E. superba* [23,82].

It was suggested that luciferin of *P. lunula* is a photo-oxidation breakdown product of the chlorophyll a [41]. LCF from any dinoflagellate can use it to produce light [40]. Based on this, Liu and Hastings [31] suggested that heterotrophic species take the luciferin nutritionally, directly or by degradation of chlorophyll from the prey. Nevertheless, the hypothesis that luciferin is originated from photo-oxidized chlorophyll [41] would only be plausible for *P. lunula*, which maintains its luciferin throughout all the daily cycle. On the other hand, fluorescent luciferin only appears in *L. polyedra* at the beginning of the night [26], so its synthesis cannot be explained by the photo-oxidation. Furthermore, *P. crassipes* (heterotrophic) can maintain its bioluminescence for a long period of time (even one year), in the absence of chlorophyll or luciferin containing food [42] and, therefore, must synthetized luciferin from another precursor. It is likely that more than one mechanism is responsible for the production of luciferin [30,38].

**Figure 10:**
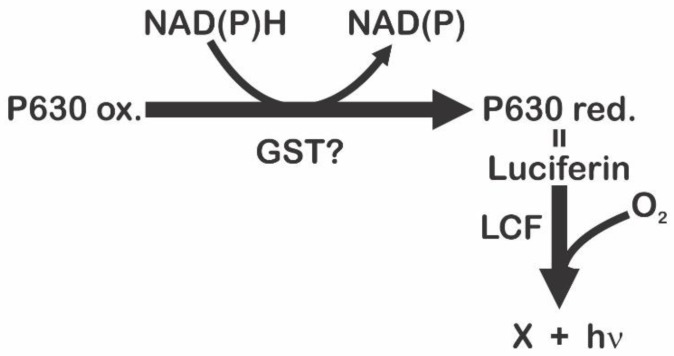
Light emission process proposed by Fresneau et al. (1986) which is controlled by at least two successive reactions, where in the first the reduction of the luciferin precursor P630 is carried out by a NAD(P)H-dependent reductase, maybe GST, and in the second, the emission of light is carried out by LCF.

In fact, using amino acids tracers, Wu et al. [43] have confirmed the intracellular production of luciferin in *P. lunula*, and Fresneau and Arrio [44] argue that bioluminescence in this species is regulated by the reduction state of the luciferin precursor. It was demonstrated that luciferin and P630, so called by his maximum excitation wavelength (630 nm), present the same peptide moiety. This is a chromo-peptide more stable than luciferin in methanol solutions at low temperature. P630 is composed of a polypeptide of 4.8 kDa, and a linear tetrapyrrole such as luciferin (600 Da). Cations may oxidize P630 or cleave the bond between the extended tetra-pyrrole and the peptide chain. Reduction of P630 could be performed enzymatically by a NAD(P)H-dependent oxidoreductase, or chemically by dithiothreitol or 2-mercaptoethanol. The state of reduction, monitored by the fluorescence and absorption emission, revealed a conformational change (pH dependent) of the chromo-peptide. These authors also report that reduced P630 has the same spectral characteristics as the purified luciferin. Furthermore, LCF can oxidize the reduced P630 with a light emission at 480 nm. It is also important to indicate that luciferin, at −20 °C on methanol, is spontaneously and partly convert into P630. This evidence points out of the interconversion P630-luciferin could be the oxide-reduction equilibrium [44]. These reports also suggest that reduced P630 is a luciferin, and the oxidized form appears to be the precursor of luciferin [83]. According to Fresneau and Arrio [44], the bioluminescent event is a complex process ruled by at least two successive reactions (Figure 10). The first is the reduction of the luciferin precursor P630 by a NAD(P)H-dependent reductase [83]. The second is the well-known LCF-luciferin reaction (Figure 1). Since P630 is reversibly reduced, seems to be an interchange point of reducing power involving a different electron transfer pathway. According with these authors, the electron transfer system regulating the reduced P630 level should be considered as the luminescence source [44]. P630 seems to be linked to complex light-modulated reactions in plant metabolism [84].

At this point is very important to note that Nakamura et al. [40] reported a product with a characteristic deep blue color during the purification process of dinoflagellate luciferin. This substance (called in this case blue compound) was isolated and purified. Nakamura et al. [40] reported that blue air-oxidation product showed UV-visible absorption maxima at 633 and 590 (shoulder) nm, suggesting the presence of a chromophore more conjugated than the found in luciferin. The UV-visible spectrum reported for the blue compound was the following: (80% methanol containing 0.1% NH_4_0Ac) 234, 254, 315, 370, 410, 590 (shoulder), and 633 nm; and the FAB mass spectrum was (glycerol) m/z 587 [(M − 2Na + 3H)+], 609 [(M − Na +H)’], and 631 [(M + H)’]. These data agree with that reported by Fresneau et al. [83] for P630: maxima absorption of P630 (oxidized) 630-370-315-250 nm; P630 (reduced) 390 nm; P630 maximum fluorescence excitation 630 nm; so, it could be suggested that the blue compound, the precursor P630, and the luciferin are different stages of the same molecule (Table 2).

According with Fresneau and Arrio [44], the bioluminescence in dinoflagellate could be thought of as a metabolic process involved in the regulation of excess intracellular reducing power produced by respiration and photosynthesis. This hypothesis is congruent with the findings of a specific chlororespiration reported on plant chloroplasts by Bennoun [85] and with other evidences found in cyanobacteria [86,87] of an alternative mechanism of respiration in photosynthetic thylakoid membranes [88].

Recently, Wang and Liu [80] suggested a mechanism of LCF catalysis in dinoflagellates linked to a Dexter energy transfer. According to these authors, the fact that luciferin, opposite to oxyluciferin, is fluorescent, with a λem very similar to the bioluminescence emission maximum, was confirmed using the Time-Dependent Density Functional Theory (TD-DFT). Based on this, it was proposed that an excited state oxyluciferin intermediate originated during the LCF catalysis could transfer energy to another molecule of luciferin, or an analog, which would then serve as the bioluminophore by relaxing with the radiative emission of light.

Nevertheless, Ngo and Mansoorabadi [89] suggested that, in dinoflagellates, an excited state intermediate derived from the reaction between O_2_ and luciferin, an excited state gem-diol(ate) intermediate, can function directly as luminophore. These authors also used the TD-DFT to study the diverse nominated LCF catalysis mechanisms. The comparison between the emission wavelength and the thermodynamic feasibility, suggest that a gem-diol(ate) intermediate as the bioluminophore, over a hydroperoxide or peroxy anion. These authors indicate that if the LCF catalytic cycle starts with the E-isomer of luciferin, which is more stable, the process probably implicate a Chemically Initiated Electron-Exchange Luminescence (CIEEL) mechanism. This process has been referring to explain other bioluminescent reactions, as in the case of fireflies [57,90,91]. However, if luciferin has the Z-configuration, the data suggest that a twisted excited state gem-diol(ate) intermediate could serve as the bioluminophore. In this case, LCF would catalyze a Twisted Intramolecular Charge Transfer (TICT) reaction. TICT states have been related on several photochemical reactions but until recently had not been linked to any bioluminescent system [89,92]. Could the P630–blue compound molecule be considered the gem-diol(ate) intermediate related with this process? This is a question that requires more investigation.

On the other hand, and as indicated above, in the photosynthetic species *P. lunula*, the luciferin is structurally similar to chlorophyll a [41], and in fact, *P. lunula* incorporated radioactively labeled chlorophyll precursors into luciferin and chlorophyll, demonstrating a link between their biosynthesis [43]. Janouskovec et al. [93] suggest that, since the non-photosynthetic species (*Oxyrrhis, Dinophysis, Noctiluca*), show cryptic plastidial metabolisms not found in the cytoplasm, all free-living dinoflagellates are dependent on plastids from a metabolic point of view. In this sence, plastid tetrapyrrole biosynthesis could explain the presence of luciferin in non-pigmented species. The three non-photosynthetic species reported by these authors carry multiple components of the plastid tetrapyrrole pathway, but only two to three components of that are present on cytosol and mitochondria. A comparison between the data obtain by Janouskovec et al. [93] and the genome of *Symbiodinium minutum*, suggest that a single tetrapyrrole pathway of a predominantly plastid origin that initiates from glutamate is present in all core dinoflagellates, a typical characteristic of eukaryotic plastids [94]. The above-mentioned non-photosynthetics species also have genes of the membrane translocators for triose phosphate, the plastid iron–sulfur system (SufB, C, D), and ferredoxin redox system (i.e., Fd NADP+ reductase FNR) [93].

According to these authors the consistent presence of signal peptides and N-terminal extensions is in harmony only with a plastid origin. The presence of the plastid tetrapyrrole pathway in the species of *Noctiluca*, leading to the precursors of chlorophyll, also could explain the production of luciferin by biosynthesis, at least in some species. Apparently, the plastid tetrapyrrole pathway is indispensable for heme synthesis in all core dinoflagellates and could therefore be related to luciferin production in any bioluminescent dinoflagellate, independently of the presence of photosynthesis. This could explain the synthesis of luciferin derived from an earlier intermediate of the chlorophyll synthesis pathway from a chlorine-like tetrapyrrole or chlorophyllide. Although this hypothesis needs to be tested, their finding of the plastid tetrapyrrole pathway reinforce the possibility that bioluminescence in non-photosynthetic dinoflagellates depend on a biosynthetic pathway readapted from chlorophyll and heme production [93].

In relation to the synthesis process of the tetrapyrrole molecule, and considering a broader context, it is evident that there is a relationship between the mechanism of synthesis of this molecule and the appearance of photosynthesis. As pointed out by Martin et al. [95], the first ecosystems on earth were chemotrophic, fueled by geological H_2_ and, for CO_2_ fixation, which required flavin-based electron bifurcation to reduce ferredoxin and it is likely that the first photochemically active pigments were Zn-tetrapyrroles. In such context, they suggest that after the evolution of red-absorbing chlorophyll-like pigments, the first mechanism of action involved a light-driven electron transport chain that reduced ferredoxin through a reaction center progenitor by electrons and H_2_S. Framed in this complex scenario, these authors also suggested that photosynthesis subsequently arose in a cyanobacterial progenitor (anoxygenic), being the chlorophyll a, the ancestral configuration [95]. It is also important to note that biosynthesis of chlorophyll a and heme, as well as its bilin pigments derived from it, share common steps and they require O2 for catalysis, and can be carried out by oxygen-dependent coproporphyrinogen III oxidase [96].

## 4. Glutathione S-Transferase (GST)

The antioxidant enzyme GST of *P. lunula* (GenBank AAN85429.1) [35,97] present a pfam05295 domain (Luciferase_N), showing also high conservation with the sequences found in LCF and LBP (Figure 8). As a possible explanation for this homology, it was suggested the exon recombination [4]. However, the role of this N-terminal sequence on *P. lunula* GST remains unknown [97]. In addition, a GST-N-Sigma-like domain (thioredoxin-like superfamily), is also detected. The members of this group can change the redox state of target proteins through the reversible oxidation of their dithiol active site, functioning as protein disulfide oxidoreductases. In the reduced state, the thiol of the cysteine is able to donate a reduction equivalent (+e-/H+) to other unstable molecules [97], such as luciferin, a highly reactive oxygen species.

GSTs comprise a large family of eukaryotic and prokaryotic isozymes known for their ability to catalyze the conjugation of the reduced form of glutathione (GSH) to xenobiotic substrates for detoxification [98,99]. Could GST be the NAD(P)H-dependent reductase that controls the state of reduction of P630? (Figure 10). The presence of this GST, with a domain of the bioluminescent system (Luciferase_N, pfam05295), which is also part of the cytochrome P450 metabolic cycle, could indicate that it is involved in the synthesis process of luciferin through a CIEEL and/or TICT electron transfer system? In *P. lunula*, the domain Luciferase_N (pfam05295) is a common thread between GST, LBP, and LCF. However, GST has not been reported in other Gonyaulacales (Figure 5).

Furthermore, studies focused on the NADPH-dependent detoxification indicated that eukaryotic cells use several mechanisms to cope with the deleterious effects of reactive carbonyls; GSTs pathway, which are associated with the GSH or thioredoxin redox cycle, conjugates aldehydes with GSH, representing an important system for detoxification [100]. The GST proteins are a diverse family; and some of these are localized in the chloroplasts and mitochondria. Because chloroplasts contain a millimolar order of GSH, the GSH-dependent detoxification mechanism is thought to be very effective [101].

An exhaustive search in the GenBank databases revealed that the presence of the domain pfam05295 (Luciferase_N) in the GST has only been reported for *P. lunula* [35,94,97]. If indeed this isoform is involved in the process of controlling the oxidative degradation of luciferin in this species, this could explain the fact that *P. lunula* contains more luciferin than any other dinoflagellate known to date, up to 100 times more than in *L. polyedrum* for example [25].

## 5. Function of Bioluminescence in Dinoflagellates

In the case of dinoflagellates, it has been proposed that defense against predators is the main ecological role of bioluminescence [102,103,104] and, therefore, would have a fundamental function in the ecosystem structure. In the marine environment, the role of bioluminescence has been studied mostly in bacteria and in the deep waters mega-fauna, where it plays several important functions like camouflage, courtship, and defense against predators [2,105]; however, the role of bioluminescence in dinoflagellates has been studied in a less detailed way and some of the concepts that have been suggested are only supported by insufficient experimental information.

It has been reported that bioluminescence mechanism of dinoflagellates can be activated by the proximity or zooplankton contact [106,107]. On the other hand, several studies have been reported the photo-phobic responses of marine zooplankton to flash of artificial light [103,108], suggesting that bioluminescence could grant an evolutionary advantage by reducing the selective predation pressure. An extensively accepted theory proposed long ago is that bioluminescence can protect indirectly by acting as a “burglar alarm”, drawing the attention of higher order visual predators to the copepod’s location [109]. It was postulated that even if the flashing dinoflagellate dies, the population will continue to benefit because the blooms are formed asexually [38]. It has been reported that copepodamides, polar lipids exuded by copepods, induce an increase in the bioluminescence of *L. polyedra*, generating a brighter flash [110]. In addition, the copepod *Acartia tonsa* goes from preferring *L. polyedra* as a prey when it does not produce bioluminescence, to an almost complete rejection when *L. polyedra* is previously treated with copepodamides to induce a greater bioluminescent capacity [111].

More recently, Hanley and Widder [112] proposed three hypotheses to explain why dinoflagellate bioluminescence deters the predation by copepod: aposematic warning, startle response, and burglar alarm. The burglar alarm is the most accepted hypothesis; however, it demands a high concentration in the bioluminescent population to be effective, therefore the bioluminescence selective advantage at lower concentrations could be the result of another role like aposematic warning or startle response.

On the other hand, Wilson and Hastings [72] have proposed another hypothesis for the original role of bioluminescence. This is based on the bioluminescence systems of fireflies and bacteria; however, it is also feasible for dinoflagellates. In this, so called “oxygen defense hypothesis”, they argue that bioluminescence systems evolved in response to low oxygen levels during the great oxidation event, when photosynthesis evolved. Since all bioluminescence systems consume oxygen, Wilson and Hastings [72] pointed out they can detoxify from reactive oxygen species like inorganic and organic peroxides, free radicals, oxygen ions, and the production of light is only a by-product. Bioluminescence would have obtained a different function when antioxidant pathways were generalized by increasing oxygen levels [38].

Nowadays, the hypothesis of defense against predators has been more investigated and established [112], and the “defense of oxygen” [72] would explain how the bioluminescence arose and evolved before it developed an effect on another group of organisms that could exert selective pressure. However, this does not implicate that bioluminescence has the same role in the modern ocean. Integrated studies of gene regulation and evolution, combined with environmental factors and ecological interactions, would provide us with new perspectives to understand the purpose of this extraordinary functional innovation in dinoflagellates [38].

## 6. Conclusions

*P. lunula* has been established as a model organism for the study of the phenomenon of bioluminescence in dinoflagellates and the presence of several isoforms of both the LCF and LBP protein in this species, highlights the variety and complexity of the underlying molecular mechanism. Likewise, the review of the available information suggests that the P630 or blue compound is one of the precursors of luciferin, and at least in *P. lunula*, the GST protein could be involved in the synthesis process of this molecule through a revolutionary electron transfer mechanism. In relation to the original ancestral function of bioluminescence in dinoflagellates, this seems to be related to a mechanism for the regulation of excess reducing power at the intracellular level, and with the passing of time it has derived in other functions, like the defense against predators, that have importance from the point of view of the structure of the ecosystem.

## Figures and Tables

**Table 1:** Accession numbers of the sequences reported in GenBank for the luciferase (LCF), luciferin binding protein (LBP) and glutathione S-transferase (GST) in the species of microalgae.

Species	LCF	LBP	GST
*Alexandrium affine*	AAV35377	AFN26992	/
*Alexandrium catenella*	/	ABY78836	/
*Alexandrium fundyense*	AEW67906	AFN26994	/
*Alexandrium monilatum*	AEW67931	AFN26995	/
*Alexandrium ostenfeldii*	AOG16037	/	/
*Alexandrium tamarense*	AAV35378	AFN27008	/
*Coratocorys horrida*	AEW67919	AFN27015	/
*Gonyaulax spinifera*	ABO61069	/	/
*Lingulodinium polyedra*	O77206	AAA29165, AAA29166	/
*Noctiluca scintillans*	AED02505	AHB24369	/
*Protoceratium reticulatum*	AAV35381	AFN27016	/
*Pyrocystis fusiformis*	AAV35379	/	/
*Pyrocystis lunula*	AAL40676, AAL40677, AAL406778	MN259726, MN259727	AAN85429
*Pyrocystis noctiluca*	AAV35380	/	/
*Pyrodinium bahamense*	KX377172	/	/
*Tripos digitatus*	AEW67915	/	/

**Table 2:** Absorption, fluorescence excitation, and emission maxima (nm) of *P630* oxidized, *P630* reduced, and luciferin on 10 mM Hepes-NaOH buffer at pH 7.2 (Fresneau et al. 1986).

Substrates	Maximum Absorption (nm)	Maximum Fluorescence Excitation (nm)	Maximum Fluorescence Emission (nm)
P630 oxidized	630-370-315-250	630	675
P630 reduced	390	390	480
Luciferin	390-250	390	480

## Abbreviations

GSH	Glutathione
GST	Glutathione S-transferase
LBP	Luciferin-Binding Protein
LCF	Luciferase
TD-DFT	Time-Dependent Density Functional Theory
TICT	Twisted Intramolecular Charge Transfer
